# ENAM gene associated with T classification and inhibits proliferation in renal clear cell carcinoma

**DOI:** 10.18632/aging.202558

**Published:** 2021-02-03

**Authors:** Xiaohan Ren, Shengjie Liang, Yang Li, Yisheng Ji, Lin Li, Chao Qin, Kai Fang

**Affiliations:** 1 Department of Urology, Shanghai Pudong Hospital, Fudan University Pudong Medical Center, Huinan, Pudong, Shanghai 201399, China; 2 The State Key Laboratory of Reproductive Medicine, Department of Urology, The First Affiliated Hospital of Nanjing Medical University, Nanjing 210029, China; 3 The First Clinical Medical College, Nanjing Medical University, Nanjing 211166, China

**Keywords:** ENAM, T classification, renal cell carcinoma

## Abstract

The potential involvement of T classification-related genes in renal clear cell carcinoma (ccRCC) must be further explored. Public data were obtained from The Cancer Genome Atlas (TCGA) database. An overall survival (OS) predictive model was developed and validated (TCGA train, 5 years, AUC = 0.73, 3 years, AUC = 0.73, 1 year, AUC = 0.76; TCGA test, 5 years, AUC = 0.74, 3 years, AUC = 0.65, 1 year, AUC = 0.73; TCGA all, 5 years, AUC = 0.72, 3 years, AUC = 0.71, 1 year, AUC = 0.75). Finally, ENAM was selected for further analysis. *In vitro* experiment indicated that ENMA is downregulated in ccRCC, and its knockdown could promote proliferation in two cancer cell lines (OSRC-2 and SW839). Immune infiltration analysis revealed that ENAM could remarkably increase the content of cytotoxic cells, NK CD56 cells, NK cells and CD8+ T cells in the tumor immune microenvironment, which may be one reason for its tumor-inhibiting effect. In summary, ENAM may suppress cell proliferation in ccRCC and can be used as a potential reference value for the relief and immunotherapy of ccRCC.

## INTRODUCTION

In the latest GLOBOCAN worldwide cancer statistics, approximately 338,000 new cases of kidney cancer have been diagnosed and 143,000 patients succumbed to this disease [1]. Renal carcinoma originates from the renal parenchymal urinary tubular epithelial system and is generally divided into four pathological subtypes: clear cell renal carcinoma (ccRCC, accounting for 70%-80%), granulosa cell renal carcinoma, mixed cell renal carcinoma, and undifferentiated cell renal carcinoma [2–4]. Despite its lowest degree of malignancy, ccRCC progression is difficult to determine due to its complexity [5] clinically. With the tumor cells originating in the renal parenchyma and easily infiltrating the renal capsule, this disease can develop into hemangioma embolus and metastasis, thereby complicating the prognosis prediction [6].

Oncologic outcomes prediction and treatment recommendation is primarily guided by the tumor-nodes-metastases (TNM) staging system. However, the definitions of TNM classification have recently been changed because clinical outcomes substantially vary even within the same stage group [7]. Finding new molecular biomarkers and designing personalized therapeutic approaches for patients with tumor have become a research focus. Zuzana Sporikova systematically summarized the clinical gene markers for triple-negative breast cancer that could serve as diagnostic and prognostic biomarkers to guide personalized therapeutic strategies [8]. A prognostic nomogram combining four immune signatures (MAL, MS4A1, OAS1, and WFDC2) with TNM stage was also developed to predict the prognosis of lung adenocarcinoma [9]. Given the benefits of genes in predicting cancer prognosis, the latest 8th edition of TNM staging system has been featured with biomarkers that are necessary for the stratification of patients requiring personalized medicine [10].

As an extraordinary technological advancement, high-throughput sequencing produces massive genomic data that can be used to understand cancer development and progression [11]. Moreover, the broad discipline of bioinformatics has become increasingly in demand due to its strengths in handling “big data”. In this study, DEGs were identified between patients with high and low T classifications. A useful model for predicting the OS of patients with ccRCC was then established. ENAM was finally selected for further analyses (gene set variation analysis, GSVA; gene set enrichment analysis, GSEA; immune infiltration analysis) and *in vitro* experiment by interacting model genes and top 20 PPI nodes. Experimental results showed that ENAM is lowly expressed in ccRCC tissues and could inhibit tumor proliferation. Therefore, this molecule may be a valuable biomarker and therapeutic target for patients with ccRCC.

## RESULTS

### Identification of DEGs in KIRC

**Figure 1:**
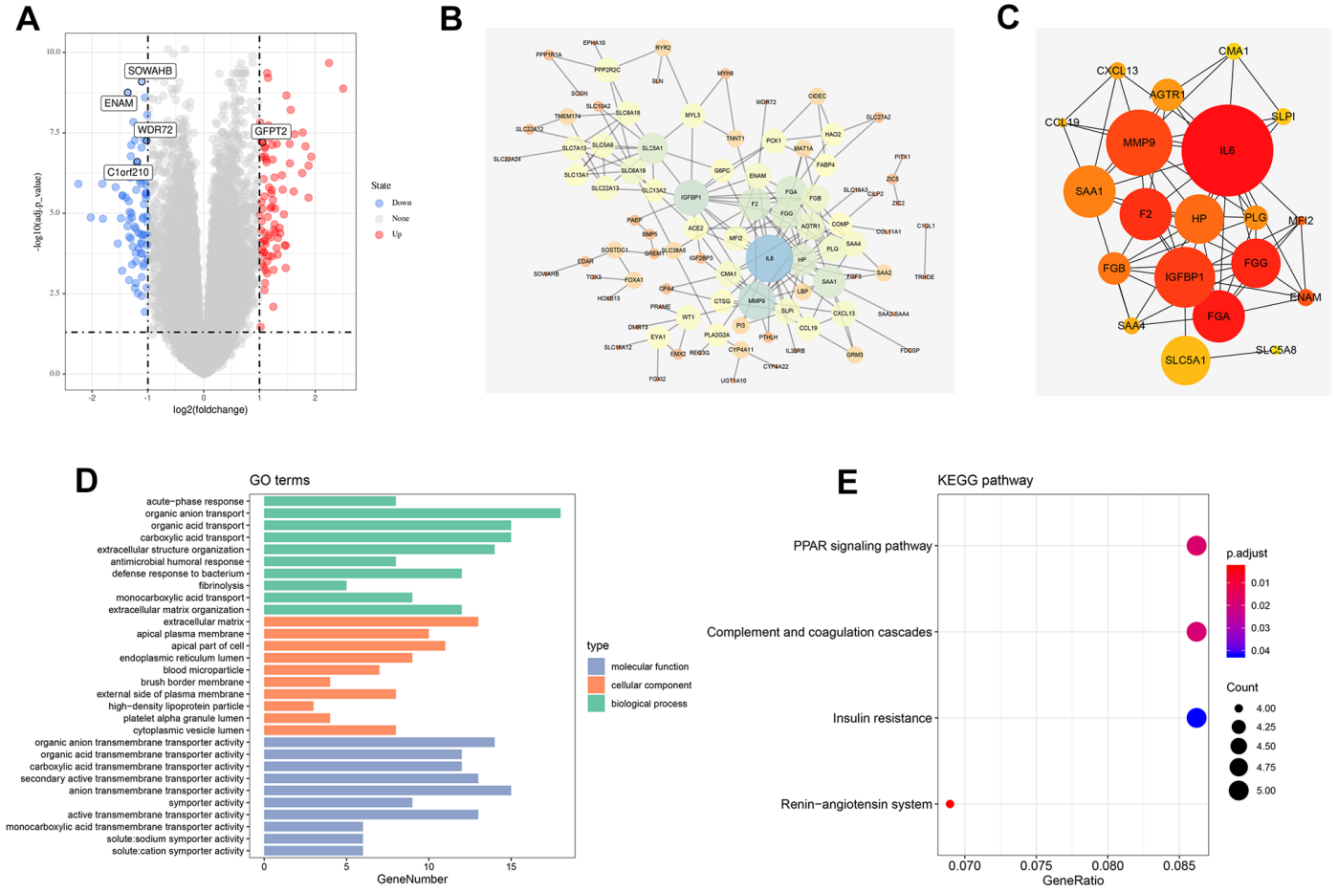
**Identification of DEGs between high and low T classification, PPI network and enrichment analysis.** (**A**) The volcano plot of TCGA; (**B**) PPI network of all DEGs; (**C**) Top 20 nodes in PPI network; (**D**) GO enrichment analysis of all DEGs; (**E**) KEGG enrichment analysis of all DEGs. Abbreviations: TCGA, The Cancer Genome Atlas; GEO, Gene Expression Omnibus; DEGs, Differentially expressed genes; PPI, protein-protein interaction; GO, Gene oncology; KEGG, Kyoto Encyclopedia of Genes and Genomes.

A total of 168 genes were differentially expressed among patients with T4 and T1 classifications with the threshold of |logFC(fold-change)|>1 and adj. P<0.05. Among which, 65 were down-regulated, and 103 were up-regulated (Figure 1A). The flowchart of the whole study was shown in Supplementary Figure 1.

### PPI network and enrichment analysis

The PPI network was visualized using the Cytoscape software (Figure 1B). MCC value was calculated by cytoHubba plug-ins that arranged all the nodes, and the top 20 crucial genes are shown in Figure 1C. GO and KEGG analysis was also performed on the identified DEGs. GO analysis revealed that for biological processes (BPs), DEGs were markedly enriched in “organic anion transport,” “organic acid transport”, “carboxylic acid transport”, and “extracellular structure organization” (Figure 1D). Changes in cellular components (CCs) were strikingly enriched in “extracellular matrix (ECM),” “apical plasma membrane,” “apical part of cell”, and “endoplasmic reticulum lumen” (Figure 1D). Changes in the DEG molecular function (MF) were primarily enriched in “organic anion transmembrane transporter activity,” “organic acid transmembrane transporter activity,” “carboxylic acid transmembrane transporter activity”, and “secondary active transmembrane transporter activity” (Figure 1D). KEGG analysis showed that DEGs were mainly enriched in “renin-angiotensin system,” “PPAR signaling pathway,” “complement and coagulation cascades”, and “insulin resistance” (Figure 1E).

### Weighted gene co-expression network (WGCNA) analysis

**Figure 2:**
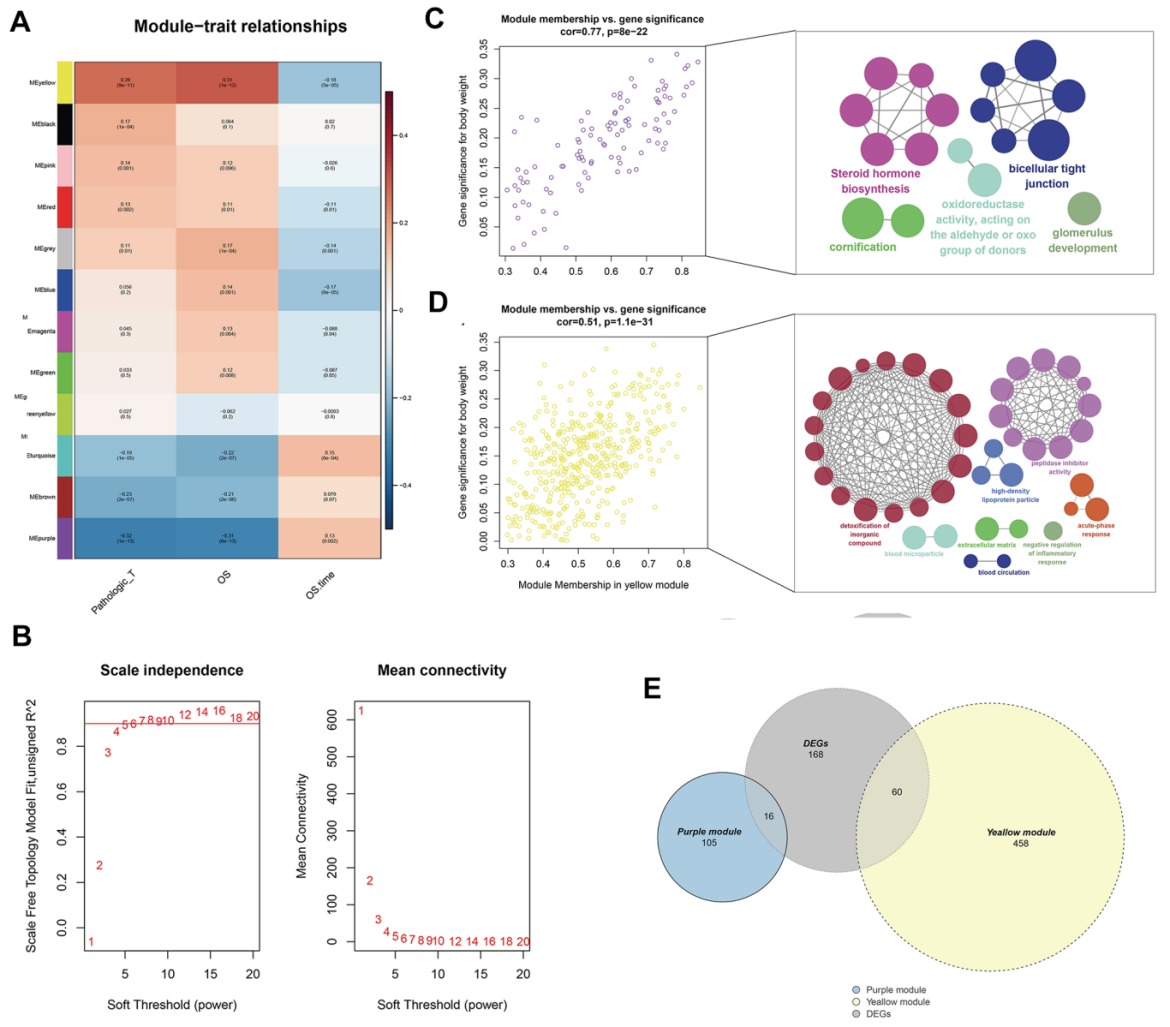
**Identification of modules associated with the T classification in the TCGA-KIRC dataset.** (**A**) Module-trait relationships. Each row corresponds to a color module and column corresponds to a clinical trait. Each cell contains the corresponding correlation and P-value; (**B**) The scale independence and mean connectivity; (**C**) The purple module genes and their GO analysis in ClueGO; (**D**) The yellow module genes and their GO analysis in ClueGO; (**E**) The venn plot of yellow module genes, purple module genes and DEGs.

WGCNA analysis was conducted on 539 tumor samples using the “WGCNA” package in R software to identify the genes associated with T classification in KIRC. “EdgeR” package was used to filter low counts, and the top 5000 differential genes were extracted. A scale-free network was constructed with the power of soft-thresholding parameter = 5 (β = 5). After modules were merged with a dissimilarity of less than 25%, 12 distinct gene modules were identified (Figures 2A, 2B). The clinical data of each sample were subsequently matched, and the correlation between each gene module and T classification was analyzed. Finally, the purple (Cor = 0.77, P <0.0001) and yellow modules (Cor = 0.51, P <0.0001) were identified as the top two having the highest correlation with T classification (Figure 2C, 2D). ClueGO analysis showed that the purple module genes were mainly enriched in the terms “steroid hormone biosynthesis” and “biocellular tight junction.” For the yellow modules, the top two terms were “detoxification of inorganic compound” and “peptidase inhibitor activity.” From the intersection of all DEGs and two modular genes, 76 genes were identified and selected for further analysis (Figure 2E).

### Establishment and validation of the OS prediction model

**Figure 3:**
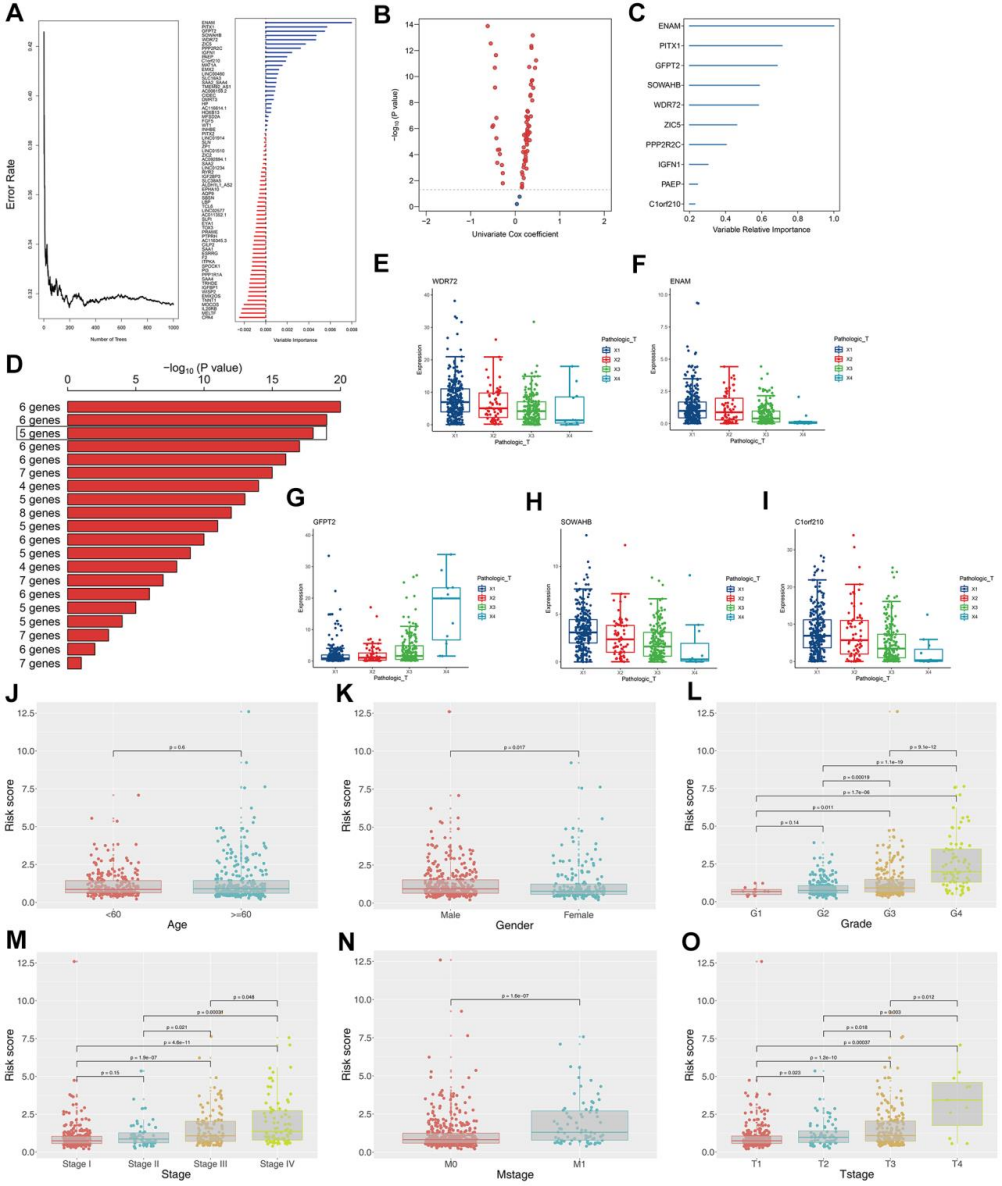
**Construction of the prognosis model based on the T classification related genes.** (**A**) Error rate for the data as a function of the classification tree and out-of-bag importance values all the predictors; (**B**) Volcano plot displayed the genes of the univariate Cox regression analysis; (**C**) Random survival forest analysis screened 10 genes; (**D**) After Kaplan–Meier analysis of 2 –1 = 1,023 combinations, the top 20 signatures were sorted according to the p value of KM. And the signature included five genes that were screened out, for it had a relative big −log10 p value and a small number of genes; (**E**) The association between WDR72 expression with T classification; (**F**) The association between ENAM expression with T classification; (**G**) The association between GFPT2 expression with T classification; (**H**) The association between SOWAHB expression with T classification; (**I**) The association between C1orf210 expression with T classification; (**J**) The correlation between risk scores and age; (**K**) The correlation between risk scores and gender; (**L**) The correlation between risk scores and grade; (**M**) The correlation between risk scores and stage; (**N**) The correlation between risk scores and Mstage; (**O**) The correlation between risk scores and Tstage.

**Figure 4:**
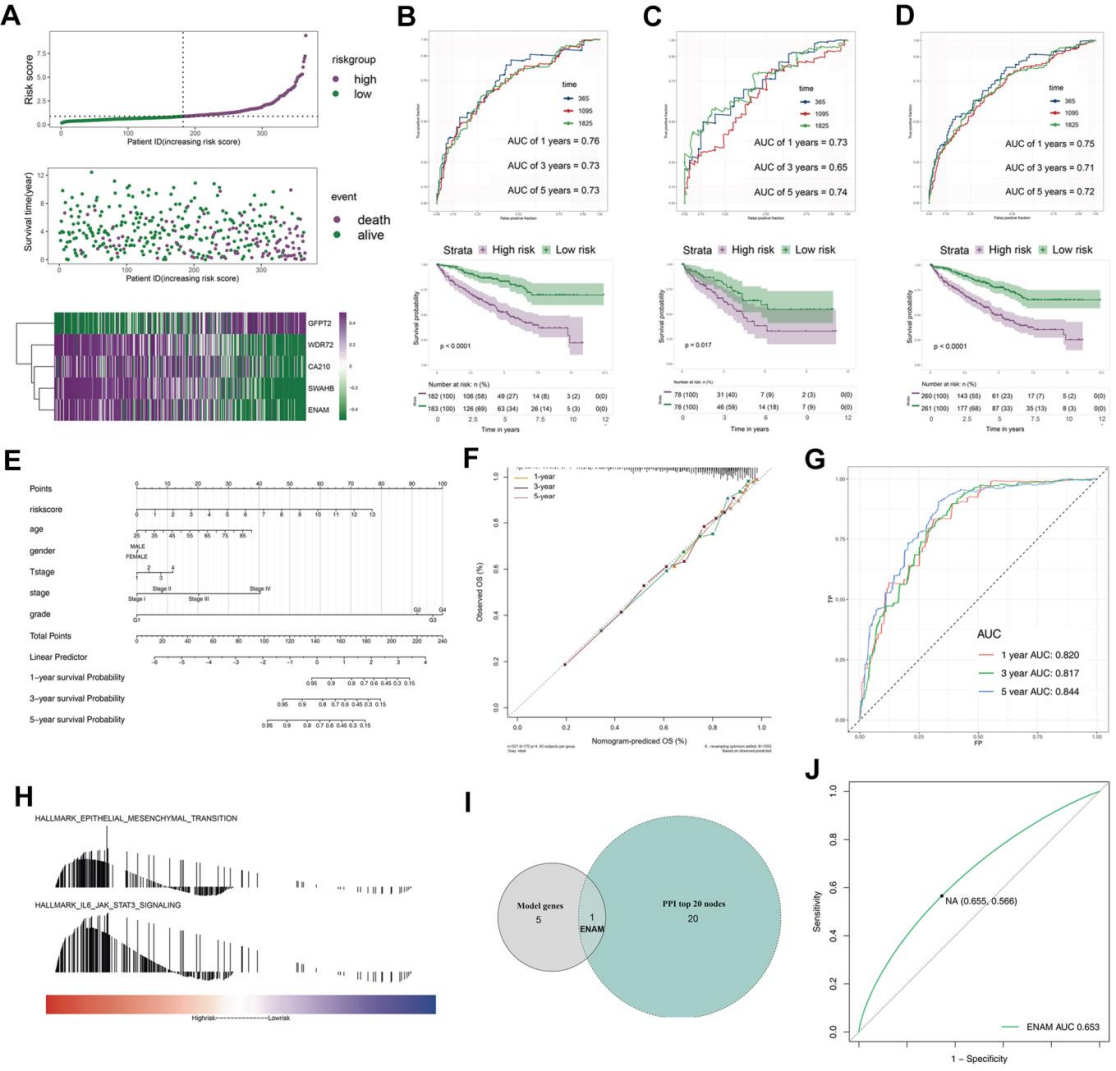
**The evaluation of the model and the nomogram plot.** (**A**) The risk plot of OS predictive model in TCGA-KIRC; (**B**) The ROC curve and Kaplan-Meier survival curves of TCGA-train group; (**C**) The ROC curve and Kaplan-Meier survival curves of TCGA-test group; (**D**) The ROC curve and Kaplan-Meier survival curves of TCGA group; (**E**) The nomogram plot; (**F**) The calibrations of 1, 3, 5 years; (**G**) The ROC curves of nomogram plot; (**H**) The GSEA analysis of high risk patients; (**I**) The venn plot of model genes and top 20 nodes in PPI; (**J**) The ROC curves of ENAM with best cutoff. Abbreviations: PPI: Protein-protein interaction.

The survival relevance of these 76 genes was further explored. Univariate cox analysis was first performed to identify prognosis-related genes (Figure 3B). Random survival forest algorithm was then used to filter the genes according to importance screening (Figure 3A). The top 10 important genes were selected for multivariate cox analysis (Figure 3C). After the permutation and combination of these 10 genes, a log rank test was used to select the combination with significant P value and less gene number, which was defined as the prognosis signature (Figure 3D). T-related genes WDR72, ENAM, GFPT2, SOWAHB, and C1orf210 were finally selected to construct a model for predicting the OS of patients with KIRC by using the formula of “Risk scores = GFPT2 expression * 0.173 + SOWAHB expression * -0.319 + ENAM expression * -0.05 + WDR72 expression * -0.081 + C1orf210 expression * 0.187” (Figure 3E–3I). The clinical correlation analysis showed that the risk scores are notably associated with worse clinicopathologic features (Figure 3J–3O; Stage III-IV, Grade 3-4, T3-4, M1). All patients were categorized into high- and low-risk groups according to their risk scores (Figure 4A). ROC curve and Kaplan–Meier survival curve revealed that the established model had good sensitivity and specificity in predicting the OS of patients with KIRC (Figure 4B, TCGA train, 5 years, AUC = 0.73, 3 years, AUC = 0.73, 1 year, AUC = 0.76; Figure 4C, TCGA test, 5 years, AUC = 0.74, 3 years, AUC = 0.65, 1 year, AUC = 0.73; Figure 4D, TCGA all, 5 years, AUC = 0.72, 3 years, AUC = 0.71, 1 year, AUC = 0.75). A multivariable cox proportional hazards model was subsequently constructed, and a nomogram was plotted based on the clinical factors and risk scores of patients in TCGA (five-genes OS prediction model). Primary clinical factors include age, gender, T classification, clinical stage, and grade (Figure 4E). The final point was the sum of the points from each item. As a continuous variable, the point of age was calculated as “0.578* age - 14.447”. For the T classification, the patients in T1, T2, T3, and T4 comprised the 0 point, 3.934 points, 7.869 points and 11.803 points, respectively. Female was the 0 point, and Male was 11.649 points. The points of Stage I, Stage II, Stage III and Stage IV was 0, 8.209, 20.275 and 40.358. As for the grade, the patients in G1, G2, G3, and G4 was 0 point, 91.727 points, 96.828 points and 100 points, respectively. The points of our predictive model was “5.923* risk scores”. The one-year survival probability was “5.08e-07* points ^3 + -0.000389964* points ^2 + 0.078786403* points - 3.834997412“. The three-year survival probability was “ 5.08e-07* points ^3 + -0.000355266* points ^2 + 0.061814757* points - 2.236972943”. The five-year survival probability was “ 5.08e-07 * points ^3 + -0.000336914 * points ^2 + 0.05347929 * points - 1.543227337“. The calibrations curves showed the great effectiveness and stability of the nomogram (Figure 4F, gray: ideal). The ROC curve of the nomogram combined with risk scores and clinical features showed greater effectiveness than the predictive model (1-year AUC, predictive model: 0.76, nomogram: 0.820, increased: 0.06; 3-year AUC, predictive model: 0.73, nomogram: 0.817, increased: 0.087; 5-year AUC, predictive model: 0.73, nomogram model: 0.844, increased: 0.114) (Figure 4G). GSEA results showed that in the high-risk group, the pathway of epithelial–mesenchymal transition and IL6-JAK-STAT3 signaling were enriched (Figure 4H). The interaction of the model genes and the top 20 PPI nodes identified only one gene—ENAM, which was selected for further analysis (Figure 4I). ROC curves revealed that the AUC value, sensitivity and specificity of ENAM in predicting OS was 0.653, 0.566 and 0.655, respectively (Figure 4J). Besides, the logistic regression analysis revealed that the impact of ENAM on patient survival is independent of patient clinical parameters (Supplementary Figure 2, P= 0.043).

### Pathway enrichment and immune infiltration

**Figure 5:**
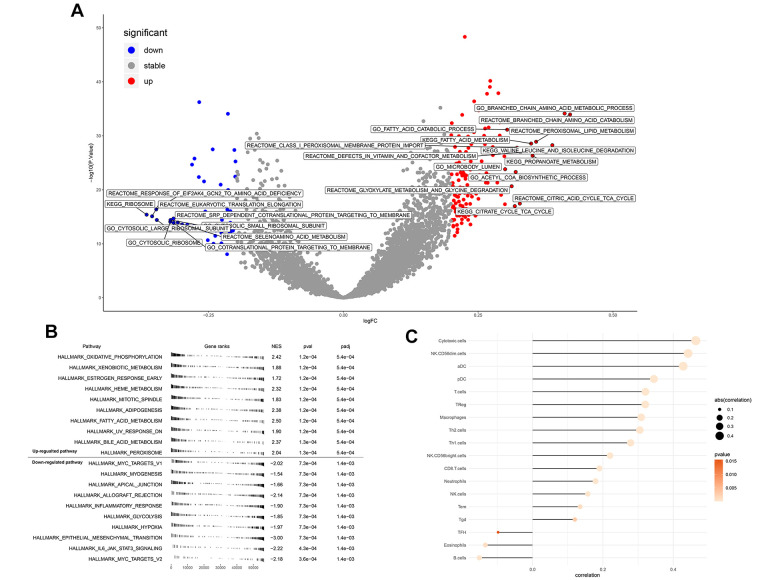
**Enrichment analysis and immune infiltration of ENAM.** (**A**) GSEA analysis of ENAM; (**B**) GSVA analysis of ENAM; (**C**) The association between KIF20A and 24 immune cells calculated by ssGSEA.

GSVA analysis determined that when ENAM expression was increased, remarkable enrichment was observed in metabolism-related pathways, such as amino acid metabolism, lipid metabolism, carbohydrate metabolism, and oxidative phosphorylation (Figure 5A and Supplementary Table 1). GSEA analysis also indicated that in high ENAM expression phenotype, the pathways of oxidative phosphorylation, xenobiotic metabolism, heme metabolism, fatty acid metabolism, and bile acid metabolism were also enriched (Figure 5B). Given the tight linage of metabolism and immunity, the underlying association between ENAM expression and immune infiltration (or multiple immune cells) was further explored. ENAM was found positively correlated with cytotoxic cells, NK CD56 cells, NK cells, CD8+ T cells, and CD8 Treg cells but negatively associated with eosinophils cells and B cells (Figure 5C).

### ENAM is down-regulated in renal cancer tissues

**Figure 6:**
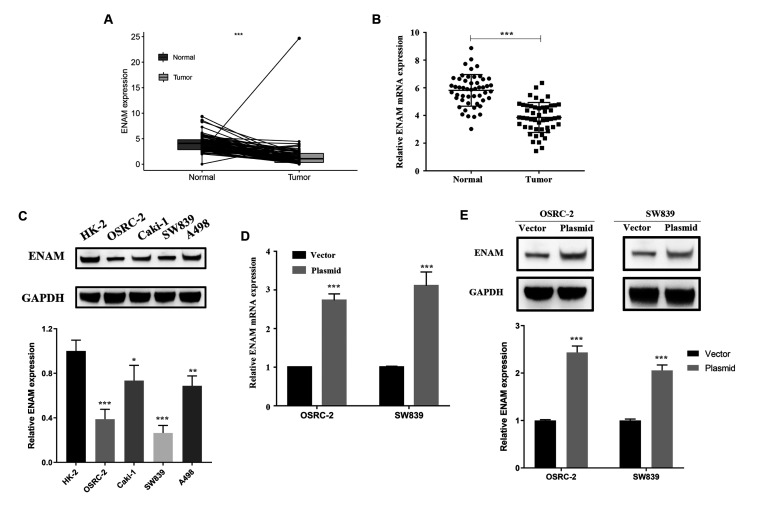
**ENAM is down-regulated in ccRCC.** (**A**) Expression of ENAM was down-regulated in 72 paired tumor compared with paratumor samples in TCGA; (**B**) Expression of ENAM was frequently down-regulated in 50 ccRCC tumor tissue; (**C**) ENAM protein expression in HK-2, OSRC-2, SW839, Caki-1 and A498 cell lines; (**D**) qPCR of indicated cells transfected with ENAM-vector and ENAM; (**E**) Western blotting of indicated cells transfected with ENAM-vector and ENAM. Abbreviations: NS: P>0.05; *: P<0.05; **: P<0.01; ***: P<0.001.

ENAM expression was evaluated in 72 paired tumor and paratumor samples, and its low expression was found in the tumor tissues (Figure 6A). Fifty pairs of ccRCC tissues were then collected for qPCR analysis to detect the actual ENAM expression pattern, and the same conclusion was obtained (Figure 6B). Western blot analysis also showed a low protein expression in kidney cancer cell lines, among which OSRC-2 and SW839 presented the lowest levels (Figure 6C). Successful ENAM overexpression *in vitro* was verified through its mRNA and protein levels by using qPCR and Western blot analysis (Figure 6D, 6E).

### Overexpressed ENMA inhibits proliferation in renal cancer

**Figure 7:**
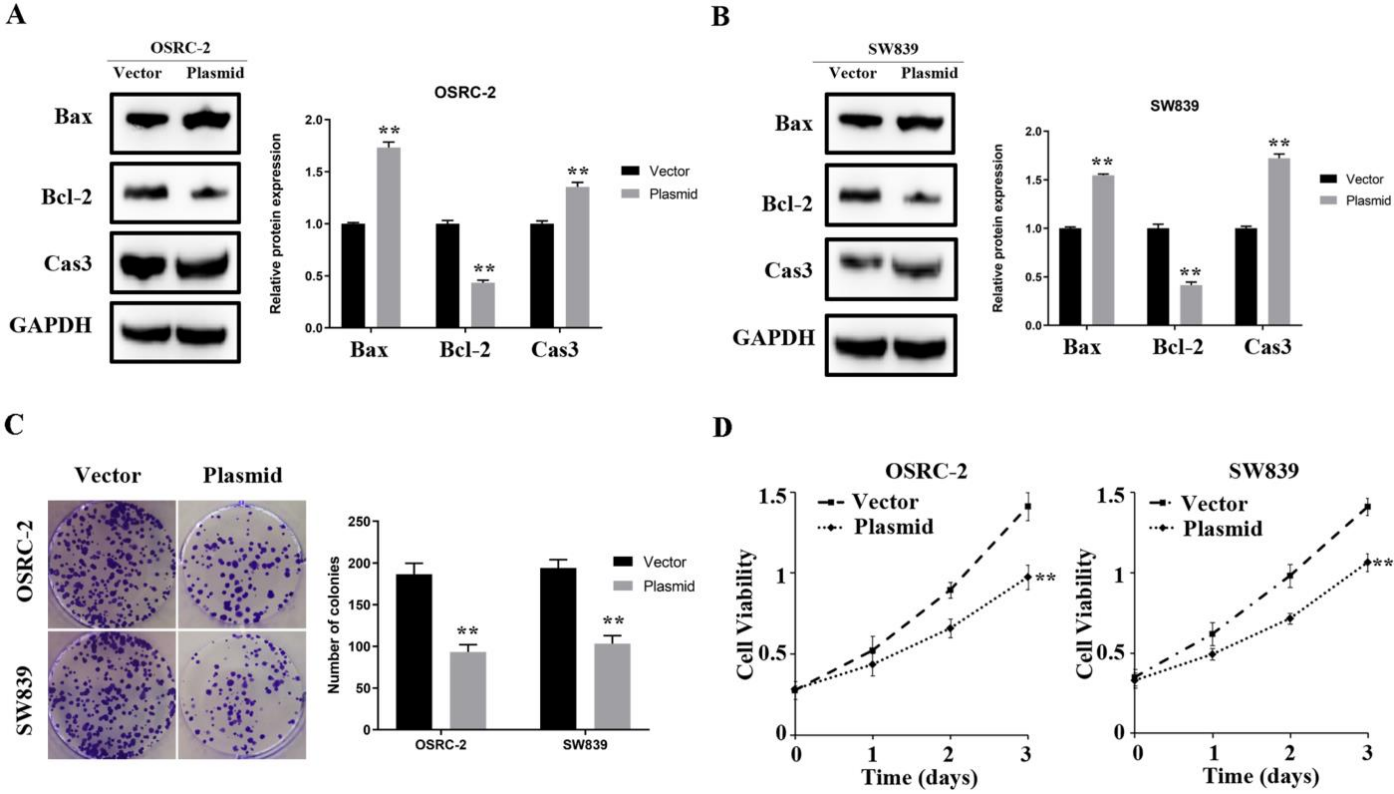
**ENAM regulates the proliferation of renal cancer cells.** (**A**, **B**) Upregulation of ENAM significantly increased the expression of Bax and Cas3, yet decreased the expression of Bcl-2; (**C**) Upregulation of ENAM reduced the mean colony number in the colony formation assay; (**D**) MTT assays revealed that upregulation of ENAM significantly reduced the cell viability. Abbreviations: NS: P>0.05; *: P<0.05; **: P<0.01; ***: P<0.001.

In OSRC-2 and SW839 cell lines, overexpressed ENAM elevated the protein level of Bax and cleaved-cas3 but decreased Bcl-2 expression (Figure 7A, 7B). Clonogenic assay showed that the overexpressed ENAM could significantly decrease clonogenic capacities (Figure 7C). MTT assays further validated this observation, indicating that ENAM overexpression could also inhibit the proliferation of renal cancer cells (Figure 7D).

## DISCUSSION

As one of the top 10 causes of cancer death, renal cancer progression is relatively difficult to predict and understand, and its control is thus challenging [12]. ccRCC is the most frequently occurring subtype of kidney cancer and causes almost over one million deaths worldwide [13]. Although the 5-year survival rate of ccRCC is above 90% at the early stage, this value declines to 15% in advance stage [13]. T classification is a powerful predictor of the condition and prognosis of patients in clinical practice. This study has considerable relevance in the search for novel biomarkers associated with T classification, new therapeutic targets, and new therapeutic methods and provides new directions for future research.

T classification-related genes remarkably associated with the OS of patients with ccRCC were screened by a series of bioinformatics analysis. These genes may participate in cancer progression, and some of which have not been reported in ccRCC. WGCNA results identified ENAM for further analysis and *in vitro* experiment. This gene was proven to inhibit proliferation in ccRCC.

A total of 145 DEGs were identified between the high T (T4) and low T (T1) staging groups on the basis of the transcriptome and clinical data of patients with ccRCC. GO enrichment analysis revealed that the gene module was predominantly enriched in some areas, including the transport of organic anion and ECM. Only a few studies focused on the transport of organic anion and extracellular matrix and were unable to clarify their vital role in cancer progression and development. Abe and their colleagues reported that OATP1B, a member of the organic anion transporting polypeptides superfamily, is up-regulated in multiple gastrointestinal cancer and associated with patient clinical outcomes [14]. A large number of unique ECM structures with multiple biological functions could multimetrically affect the physiological and biochemical processes of cells [15]. Along with tumor progression, the increase in structural constituents such as ECM in the tumor microenvironment could lead to rapid tumor growth [16, 17].

A model with five genes was constructed for predicting the OS of patients with ccRCC on the basis of all the DEGs and two modular genes of WGCNA. KM and ROC curve revealed that the model was reliable for the training and validation groups. Furthermore, GSEA analysis was conducted to understand the biological difference between the high- and low-risk groups. The results suggested that the epithelial–mesenchymal transition (EMT) and IL6-JAK-STAT3 signaling were the top two significant pathways that play an essential role in the progression and development of multiple tumors [18, 19]. EMT is a process in which epithelial cells transdifferentiate into motile mesenchymal cells and obtain the capacity of cell movement [20]. By using the transgenic mouse model, Zeisberg and their colleagues revealed that the EMT might be an essential source for the carcinoma-associated fibroblasts and critically contributes to tumor progression [21]. EMT plays a vital role in the development and progression of multiple tumors [22]. By exploring the B7-H4 level in glioma tissues with different grades, Yao and their colleagues found that the cross-talk between glioma-initiating cells and macrophages mediated by B7-H4 through the IL6/JAK/STAT3 pathway could result in poor prognosis [23]. In hepatocellular carcinoma, miR-515-5p inhibits the invasion and migration of cancer cells by suppressing the IL6/JAK/STAT3 pathway [24].

Intersection with five model genes and PPI top 20 genes revealed that ENAM might be significantly correlated with T classification and patient prognosis. This gene encodes the largest protein in the enamel matrix of developing teeth and is generally associated with dental caries [25]. However, in some tumors, ENAM exhibits extraordinary expression patterns and is possibly involved in the disease progression. For instance, Sanchez and their colleagues identified the overexpression character of ENMA based on a cohort of 100 B-cell precursors from patients with acute lymphoblastic leukemia; this feature may lead to poor prognosis [26]. To date, the role of ENAM in ccRCC has not been reported. In this work, a series of *in vitro* experiments revealed that ENAM is down-regulated in high T classification ccRCC and could inhibit the proliferation of ccRCC cells.

Immune infiltration analysis showed a positive correlation between ENAM expression and multiple immune cells such as cytotoxic cells, NK CD56 cells, NK cells, and CD8^+^ T cells, et al. This finding partially explains the tumor-suppressing function of ENAM. According to the comprehensive systematic review conducted by Martínez–Lostao, cytotoxic and NK cells are chief participants in killing tumor cells [27]. Dumont and their colleagues found that CD8^+^ T cells have a high expression of cytotoxic molecules that could effectively inhibit the proliferation of cancer cells and thus may be a target for immune therapy [28]. These results indicated that ENAM might inhibit the occurrence and development of cancer through its interactions with these immune cells and therefore has a potential reference value for the relief and immunotherapy in ccRCC.

This study inevitably suffers from certain limitations. Although the data were based on a large sample in the TCGA database, the small number of included Asian patients remains to be a limitation. Whether the conclusion applies to patients with ccRCC in Asia remains unclear. In addition, the clinical data stored in TCGA-KIRC were limited. As a result, the T classification data used for analysis were not comprehensive and may lead to potential errors or biases. Finally, the mechanism by which ENAM inhibits the progression of ccRCC requires further investigation.

## CONCLUSIONS

An effective predictive model for OS in ccRCC based on five T classification related genes was established by conducting serial bioinformatics analysis and *in vitro* experiment. ENAM was found to inhibit proliferation in ccRCC, which has not been previously reported. Its interaction with immune cells in the tumor microenvironment renders ENAM as an underlying immunotherapy target in ccRCC.

## MATERIALS AND METHODS

### Acquisition of public data and preprocessing

The gene expression profile of patients with ccRCC were obtained from TCGA database (TCGA-KIRC, FPKM), the world’s largest resource integrating the genomic and clinical information of 33 cancers [29]. Clinical and prognosis information was downloaded as “bcr xml” file and processed by the author’s own R code. Data preprocessing included background correction, data normalization, complementing missing values, and combining normal and tumor group data. “Homo_sapiens.GRCh38.99.chr.gtf” file was used for ID transformation.

### DEG identification and enrichment analysis

R package “limma” stored in Bioconductor was used to identify the DEGs between T4 and T1 tissues with the threshold of |log^FC(fold-change^)|>1 and adj. P<0.05 [30]. GO and KEGG analyses were performed using the “ClusterProfiler” package for the underlying function of DEGs [31].

### PPI network construction

The protein interaction information of identified DEGs was obtained from STRING (http://string-db.org; Search Tool for the Retrieval of Interacting Genes), an online biological database used to excavate key regulatory genes [32]. The meaning of network edges was based on evidence, and protein interaction with a score of >0.4 was considered statistically significant. Plug-ins “Cytohubba” was used to identify the hub genes according to the MCC value [33]. Open-source bioinformatics software, Cytoscape (version 3.4) was used to visualize PPI networks [34].

### Weighted correlation network analysis (WGCNA)

A co-expression network was constructed using the WGCNA package to identify the significant mRNAs associated with the T classification in ccRCC [35]. The goodSamplesGenes function was applied to check whether the DEmRNAs of data matrix meet the criteria and to eliminate the unqualified data. The pickSoftThreshold function was used to calculate the value of β (a soft threshold power parameter) to ensure a scale-free network. A tree diagram was also visualized by hierarchical clustering. The correlation between module eigengenes (MEs) and clinical traits was calculated and used to screen the MEs related to the T classification of ccRCC.

### Construction of predictive-model and nomogram for predicting OS

All candidate genes were first analyzed by univariate cox regression analysis to identify prognosis-related genes. Supervised regression random forests were then performed using the R package “randomForestSRC” for dimension reduction (ntree = 1000). The top 10 significant genes were selected for multivariate cox regression analysis. The prognosis model was established with “Risk scores = ∑coef * Exp(genes) On the basis of the clinical features and risk scores, a nomogram was established for predicting the OS of patients with ccRCC and evaluated using calibration plots.

### Pathway enrichment analysis

GSVA analysis was performed by the GSVA package in R software, a gene set enrichment method estimating the variation of pathway activity across different samples in an unsupervised manner [36]. GSEA was conducted [37] with the following parameters to evaluate the biological characteristics: “collapse data set to gene symbols” was false; the number of permutations was 1000; the “Collapse/Remap to gene symbols” was No_Collapse; the cut-off criteria was FDR <0.25 and nominal P-value <0.05; and the metric for ranking genes was Signal2Noise. The high expression group was regarded as the experimental group, and the low expression group was set as a reference group. “h2.all.v7.2.symbol.gmt (Hallmarks)” gene set database was selected for enrichment analysis.

### Immune infiltration analysis

ssGSEA package was used to quantify the content of immune cells in TCGA samples. Its advantage is the high degree of freedom in quantification. Information for the maker genes in 24 immune cells was obtained from Bindea et al. [38].

### Cell lines and qPCR

Tissues and informed consent were obtained from Pathology Tissue Bank of Jiangsu Province Hospital. Human kidney cancer cell lines (OSRC-2, SW839, Caki-1, and A498) and normal HK-2 cells were obtained from iCell (Shanghai, China). Total RNA was isolated using Trizol (Invitrogen, USA). PrimeScript RT Master Mix (Takara, JPN) was used for cDNA synthesis. qPCR was performed using an SYBR Green assay for the analysis of ENAM mRNA expression following the manufacturer’ s instructions (Applied Biosystems, USA). The primers used were as follows: ENAM, forward: 5′-GGCTTCTTGGTAATTCTGTTGCT-3′; ENAM, reverse: 5′-ATGTGGGCCGTTCATAAAGTT-3′; GAPDH, forward: 5′-AC CACAGTCCATGCCATCAC-3′; GAPDH, and reverse: 5′-TCCACCACCCTG TTGCTGTA-3′.

### Protein extraction and western blot

Total proteins were extracted from kidney cancer tissues with Western and IP lysis buffer (Beyotime, P0013; Beijing, China). Protein concentration was measured using the BCA reagent kit (Pierce, 23227, USA). The proteins were resolved by 8%–12% SDS-PAGE and then blotted onto polyvinylidene fluoride (PVDF) membranes, which were then blocked in TBS/0.1% Tween-20 (TBST) containing 5% skimmed milk powder for 1 h at room temperature. Primary ENAM, Bax, Bcl-2, cleaved-casp3, and GAPDH antibodies were diluted with 1:300 (AtaGenix, Wuhan, China) and 1:2000 (AtaGenix, Wuhan, China) prior to incubation for 2 h at room temperature. The secondary antibody [anti-rabbit or anti-mouse IgG (H+L) biotinylated antibody (CST, USA)] was incubated for 2 h at room temperature (RT).

### Vectors transfection

ENAM cDNA fragments were prepared using the EcoRV/Xhol double-enzyme digestion method. The cDNA sequences of ENMA were cloned into the pcDNA3.1 vectors to generate overexpressed ENAM (OE-ENAM).

### MTT assay

Cells were seeded into a 96-well plate at the concentration of 2×10^3^ cells/well in triplicate and then treated with 100 μl of 0.5 mg/ml sterile MTT for 4 h (37° C, 5% CO2; 24 h, 48 h, and 72 h). The medium was then removed, and 150 μl of dimethyl sulfoxide was added. Cell viability was determined by MTT assay.

### Clonogenic assay

The cancer cell lines were transfected with the above vectors for 24 h. The cells were plated into 30 mm cell culture dishes containing 10% FBS and cultured for 14 days. The medium was changed every 3 days. The cells were fixed with 15% formaldehyde for 15 min and stained with 0.1% crystal violet for 20 min prior to counting.

### Statistical analysis

Software R v3.6.1, SPSS v23, and ImageJ were used for all the analyses. All statistical tests were two-sided. P-value <0.05 was considered statistically significant. All experiments were performed at least three times. Average linkage method and Pearson correlation analysis were applied in the WGCNA analysis.

## Supplementary Material

Supplementary Figures

Supplementary Table 1
