# Supplementary material for: ENAM gene associated with T classification and inhibits proliferation in renal clear cell carcinoma
Source: Aging (Albany NY). 2021 Feb 3;13(5):7035–51. doi: 10.18632/aging.202558 (PMC7993715; doi:10.18632/aging.202558)
Supplement: Supplementary Figures [file aging-13-202558-s001.pdf]

SUPPLEMENTARY FIGURES

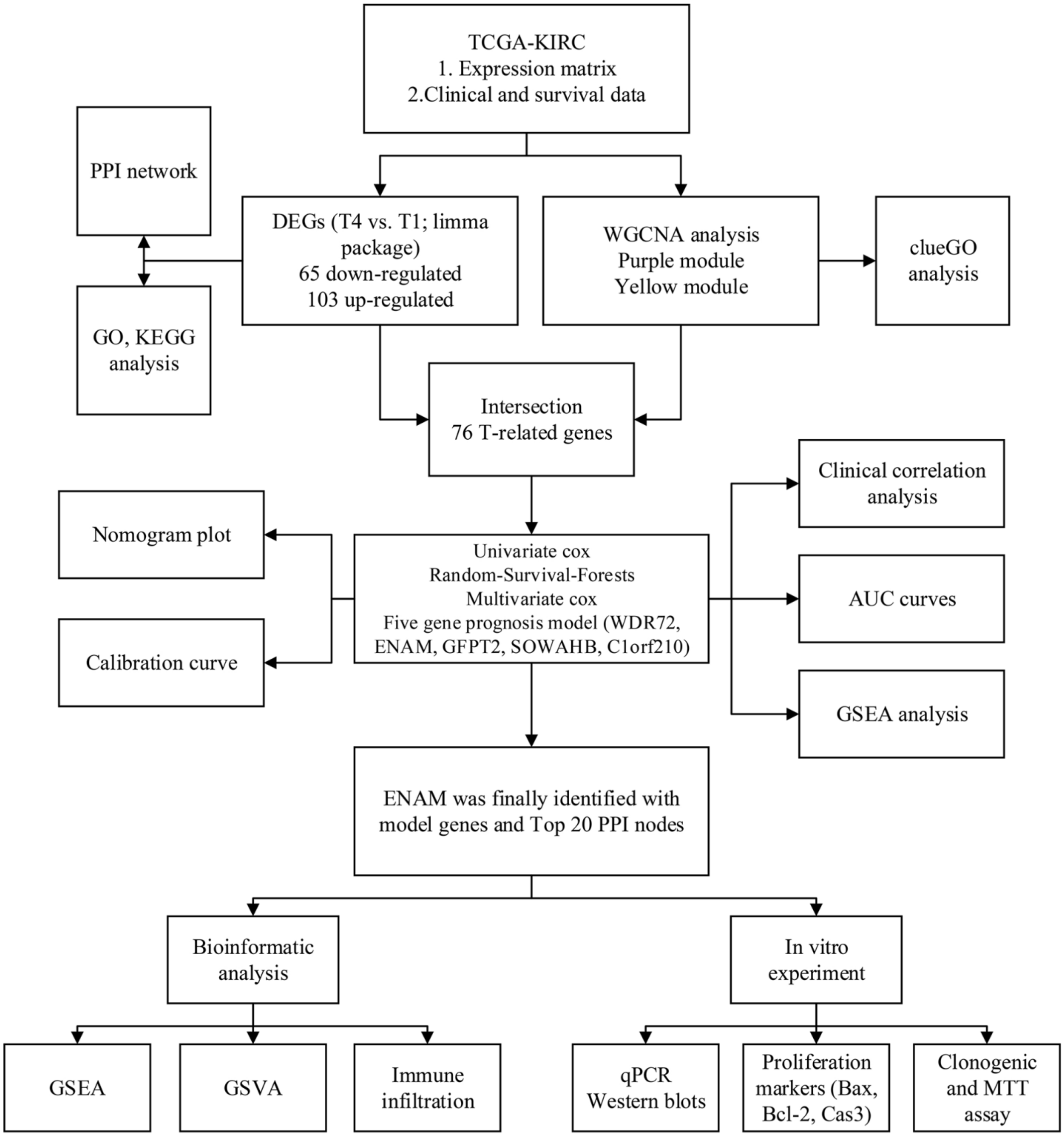

Supplementary Figure 1. The flowchart of the whole study.

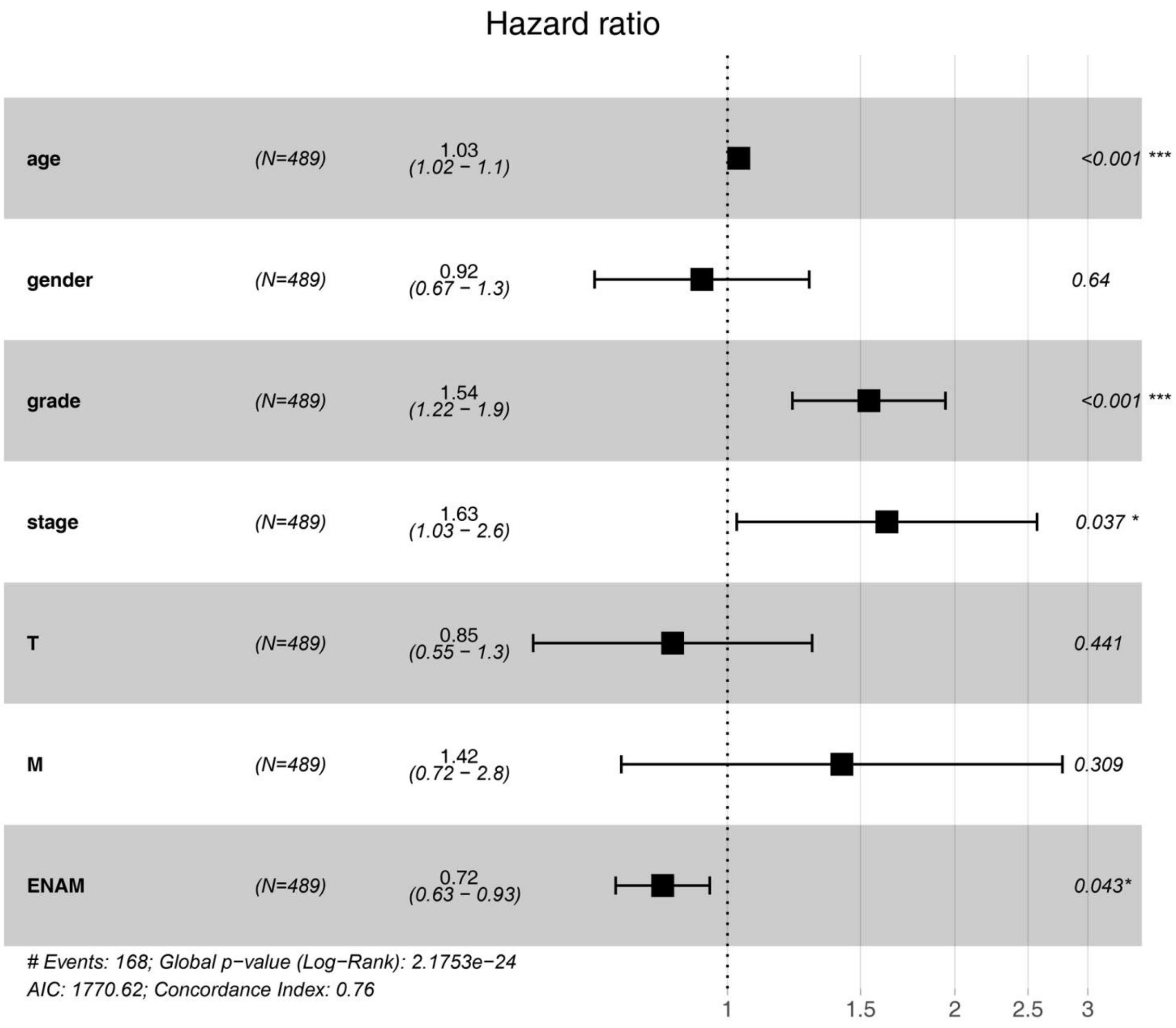

Supplementary Figure 2. Logistic regressive analysis including ENAM and clinical features on patient survival.
