# Supplementary material for: ENAM gene associated with T classification and inhibits proliferation in renal clear cell carcinoma
Source: Aging (Albany NY). 2021 Feb 3;13(5):7035–51. doi: 10.18632/aging.202558 (PMC7993715; doi:10.18632/aging.202558)
Supplement: Supplementary Table 1 [file aging-13-202558-s002.pdf]

SUPPLEMENTARY TABLE

Supplementary Table 1. The top 20 significant pathways of GSVA analysis (ENAM).

Term	logFC	P.Value	adj.P.Val
**Top 10 up-regulated pathway**			
REACTOME_BRANCHED_CHAIN_AMINO_ACID_CATABOLISM	0.42	1.10E-34	5.05E-32
GO_BRANCHED_CHAIN_AMINO_ACID_METABOLIC_PROCESS	0.41	7.24E-35	3.98E-32
KEGG_VALINE_LEUCINE_AND_ISOLEUCINE_DEGRADATION	0.39	5.27E-29	5.80E-27
REACTOME_PEROXISOMAL_LIPID_METABOLISM	0.36	1.24E-29	1.59E-27
KEGG_PROPANOATE_METABOLISM	0.35	4.81E-27	3.44E-25
KEGG_FATTY_ACID_METABOLISM	0.35	2.66E-29	3.12E-27
REACTOME_DEFECTS_IN_VITAMIN_AND_COFACTOR_METABOLISM	0.34	9.02E-28	7.29E-26
REACTOME_CITRIC_ACID_CYCLE_TCA_CYCLE	0.32	3.48E-18	6.13E-17
REACTOME_CLASS_I_PEROXISOMAL_MEMBRANE_PROTEIN_IMPORT	0.32	1.57E-28	1.57E-26
GO_ACETYL_COA_BIOSYNTHETIC_PROCESS	0.32	4.85E-24	2.26E-22
**Top 10 down-regulated pathway**			
KEGG_RIBOSOME	-0.36	3.93E-16	4.89E-15
REACTOME_EUKARYOTIC_TRANSLATION_ELONGATION	-0.35	7.78E-16	9.07E-15
REACTOME_RESPONSE_OF_EIF2AK4_GCN2_TO_AMINO_ACID_DEFICIENCY	-0.35	4.09E-17	5.99E-16
GO_CYTOSOLIC_LARGE_RIBOSOMAL_SUBUNIT	-0.35	3.80E-15	4.05E-14
GO_CYTOSOLIC_RIBOSOME	-0.32	8.21E-15	8.45E-14
GO_CYTOSOLIC_SMALL_RIBOSOMAL_SUBUNIT	-0.32	3.77E-15	4.03E-14
REACTOME_SRP_DEPENDENT_COTRANSLATIONAL_PROTEIN_TARGETING_TO_MEMBRANE	-0.32	2.08E-15	2.29E-14
GO_COTRANSLATIONAL_PROTEIN_TARGETING_TO_MEMBRANE	-0.31	1.24E-14	1.24E-13
REACTOME_SELENOAMINO_ACID_METABOLISM	-0.31	1.11E-14	1.12E-13
REACTOME_EUKARYOTIC_TRANSLATION_INITIATION	-0.30	2.62E-14	2.50E-13
